# Recalibrating stress regulation through mindfulness: a framework for adapting interventions for ACE-affected populations

**DOI:** 10.3389/fpsyg.2026.1801095

**Published:** 2026-04-10

**Authors:** Natalie R. Beylin, Matthew J. Zawadzki

**Affiliations:** Department of Psychological Sciences, University of California, Merced, Merced, CA, United States

**Keywords:** adverse childhood experience, mind–body approach, mindfulness, mindfulness-based intervention, stress regulation, trauma-informed approach

## Abstract

Mindfulness-based interventions (MBIs) are widely used to promote psychological and physical health, yet their efficacy among individuals with histories of adverse childhood experiences (ACEs) has been mixed. While most conceptualizations attribute the cultivation of nonjudgmental awareness as a primary mechanism of MBI-related benefits, individuals with stress-regulatory systems that were shaped by early life stress may have trouble engaging with such practices. To address this, this paper proposes the Mindfulness Recalibration Model (MRM), a theoretically grounded framework that reconceptualizes mindfulness practice as an experience-dependent process of biopsychological recalibration rather than solely the acquisition of cognitive-affective skills. The MRM highlights the distinct yet complementary roles of somatic mindfulness practices, which directly engage physiological regulation primarily through a bottom-up pathway, and cognitive-affective mindfulness practices, which support attentional and regulatory control primarily through a top-down pathway, and emphasizes how their coordinated and reciprocal engagement reshapes stress-regulatory systems. The model further emphasizes the iterative and self-reinforcing nature of this recalibration process across temporal levels. By centering stress-system recalibration as the primary target of mindfulness practice for individuals with histories of ACEs, the MRM offers a unifying explanation for mixed findings in this literature and provides a conceptual roadmap for the development of interventions aligned with adversity-shaped regulatory systems.

## 1 Introduction

Over the past two decades, mindfulness-based interventions (MBIs) have gained substantial popularity across a broad range of clinical and health contexts (Zhang et al., 2021a), particularly as a tool to reduce stress and improve stress-related psychological and physical health outcomes (de Vibe et al., 2012). As a psychological construct, mindfulness is commonly defined as present-moment awareness characterized by an accepting, nonjudgmental stance and reduced reactivity toward internal and external experiences (Brown and Ryan, 2003). Consistent with this conceptualization, MBIs are typically designed to cultivate these capacities through cognitive-affective practices that emphasize attentional monitoring, acceptance, and decentering from external and internal experiences as core therapeutic mechanisms (Gu et al., 2015; Shapero et al., 2018; Baer et al., 2019). Although most standardized MBIs also include somatic practices (e.g., breathing practices, body scans, gentle movement), intervention effects are frequently attributed broadly to the development of mindfulness as a cognitive-affective capacity, with less explicit attention to how different practices may engage distinct regulatory pathways.

A growing body of research has examined whether these approaches may also benefit individuals with histories of early life stress, or adverse childhood experiences (ACEs; Moyes et al., 2022). To date, findings in this area have been mixed. MBIs that emphasize acceptance and decentering from distressing internal experiences have shown promise for improving psychological functioning among some individuals with ACE histories (Spidel et al., 2018), however, these effects were not consistently replicated (Spidel et al., 2019). This lack of replication is not surprising as interventions for those who have experienced early life adversity tend to show inconsistent efficacy across varying psychological outcomes (Kirlic et al., 2020; Moyes et al., 2022). Moreover, this literature is further complicated by the common practice of failing to measure and/or report negative or adverse outcomes (Moyes et al., 2022). Notably, recent work highlights how mindfulness-based approaches may carry iatrogenic risk for some ACE-exposed individuals, with negative outcomes varying as a function of trauma type, delivery format, or participant characteristics (Lindahl et al., 2017; Canby et al., 2025). For individuals whose stress-regulatory systems have been shaped by early life adversity, cultivating a sustained attention to internal experience with nonjudgmental awareness may be particularly difficult to access or sustain. This raises a critical conceptual question regarding how mindfulness practices should be structured and sequenced for ACE-affected populations, given that the very regulatory capacities they aim to cultivate are often those most disrupted by early adversity.

Taken together, a framework is needed to further clarify the mechanisms of MBIs, particularly for those with a history of ACEs, to optimize these interventions for addressing stress-related processes and outcomes. Therefore, the present paper introduces the Mindfulness Recalibration Model (MRM), a theoretically grounded framework that conceptualizes mindfulness practice as an experience-dependent process of stress-system recalibration, rather than solely the acquisition of cognitive-affective skills. The MRM proposes that, whether intentionally or not, MBIs well-suited to address the needs of ACE-affected individuals operate through the coordinated engagement of bottom-up physiological pathways and top-down cognitive-affective pathways via both somatic and cognitive-affective mindfulness practices, respectively. In contrast, interventions that focus primarily on cognitive-affective mindfulness practices may fail to engage the foundational bottom-up pathways, thereby limiting their effectiveness or durability for individuals with ACE histories. To date, much of the research examining MBIs has assessed physiological and psychological outcomes separately (Van Dam et al., 2018), or has yielded inconsistent results across these domains (Baumgartner and Schneider, 2023). This siloed approach may obscure the integrated, multi-system nature of stress regulation and limit understanding of how MBIs produce their effects, especially for ACE-affected populations.

While a wide range of evidence-based treatments for trauma-exposed populations have demonstrated efficacy in targeting stress-related symptoms through multi-component strategies (Schnyder et al., 2015; Coventry et al., 2020), mindfulness-based approaches represent a rapidly emerging component within this broader landscape that remains less well understood. Accordingly, we frame the MRM not as a replacement for established trauma treatments, but as a framework for optimizing the role of MBIs within broader integrative approaches to chronic stress and trauma recovery. As the present framework elaborates, this requires a more mechanistically precise account of how MBIs engage both bottom-up physiological and top-down cognitive-affective pathways, and how their coordinated interaction supports regulatory change over time.

## 2 Rethinking mindfulness-based interventions for ACE-affected populations

Mindfulness is broadly defined as the intentional, nonjudgmental awareness of present-moment experience (Brown et al., 2007). Traditional conceptual accounts of how mindfulness training works suggest that MBIs strengthen coping and mental health by helping people become more aware of, and better manage, negative thoughts and emotions (Baer et al., 2019). In line with this perspective, many interventions are designed to cultivate a ‘mindful state’ (Zhang et al., 2021b), which, when repeated over time, gradually strengthen trait-level mindfulness (Kiken et al., 2015). This process involves teaching individuals to sustain attention to the present-moment, tolerate distress without disengaging, decenter from internal experience, and flexibly modulate cognitive and emotional responses (Shapiro et al., 2006; Gu et al., 2015; Shapero et al., 2018). In addition to these cognitive-affective practices, most standardized MBIs also include a range of somatic practices. Some of these remain somewhat cognitive in nature, such as focused attention to the breath or bodily sensations, whereas others involve more explicitly body-focused engagement, including breathing exercises, gentle yoga, and mindful movement (de Vibe et al., 2012; Santorelli et al., 2017). However, because mindfulness as a construct is most often conceptualized in terms of attentional and cognitive-affective components (Malinowski, 2013), the somatic practices within MBIs may be overlooked or treated as peripheral when evaluating intervention mechanisms. As such, observed effects are frequently attributed broadly to “mindfulness training” and the practice of cultivating mindfulness itself (Zhang et al., 2021b), rather than to the specific cognitive-affective and somatic practices that comprise these interventions and may differentially contribute to regulatory change.

This disconnect between how mindfulness is conceptualized, the content of MBIs, and their proposed mechanisms is particularly consequential for ACE-affected populations. When the primary goal of intervention is framed as cultivating mindfulness itself, programs may be designed and evaluated to primarily focus on the cognitive-affective practices with somatic practices treated as brief, introductory, or secondary. However, individuals who have experienced early adversity may not access the cognitive-affective practices, or develop the core cognitive-affective components of mindfulness, in the same way. Given that early stress can shape stress-regulatory systems toward heightened threat vigilance and survival-oriented responding (Danese and McEwen, 2012; McLaughlin et al., 2014, 2016), the attentional and inhibitory control capacities particularly targeted by cognitive-affective mindfulness practices may be difficult to engage or sustain (Arnsten, 2009; Sheffler et al., 2020).

### 2.1 ACEs impact on top-down cognitive and affective regulation

ACEs, including abuse, neglect, and household instability, are associated with enduring alterations in top-down regulatory systems that support attentional control, emotion regulation, and flexible responding to stress. Developmental exposure to chronic or unpredictable stress interferes with the maturation and functioning of prefrontal cortical regions responsible for orchestrating executive control, cognitive flexibility, and reflective emotion regulation (Danese and McEwen, 2012). As such, ACEs are often associated with entrenched patterns of maladaptive cognitive and emotion regulation patterns, such as self-judgment, disengagement, suppression, or perseverative thinking (Gunduz et al., 2019; Sheffler et al., 2019; Sheffler et al., 2020).

These entrenched traits are directly at odds with mindfulness practices involving a nonjudgmental awareness of the present moment. For instance, prior work has found that elevated perseverative cognitions can inhibit engagement with mindfulness practices (Banerjee et al., 2018). Moreover, reduced prefrontal modulation of limbic regions contributes to heightened amygdala responsivity and a tendency towards rapid threat-processing, even in relatively neutral situations (Fareri and Tottenham, 2016; McLaughlin et al., 2016; Nusslock and Miller, 2016). Thus, rather than fostering receptive attention, ACE-related adaptations often bias attention toward internal distress signals or external threat cues. Taken together, individuals with histories of ACEs may have a particularly difficult time intentionally directing attention towards present moment experiences with a non-judgmental attitude, which are central capacities of cognitive-affective mindfulness practices. Alternatively, focused attention on internal distress signals may prompt avoidance or disengagement, rather than a nonjudgmental awareness (Zhu et al., 2019; Canby et al., 2025).

### 2.2 ACEs impact on bottom-up physiological regulation

ACEs exert significant effects on bottom-up physiological stress systems, particularly the autonomic nervous system (ANS) and hypothalamic–pituitary–adrenal (HPA) axis, which set the stage for the top-down alterations discussed above. The ANS governs the body’s ability to shift between states of arousal as needed. However, repeated stressful or traumatic experiences in early life bias the ANS toward frequent activation of the sympathetic nervous system (SNS), the body’s “fight-or-flight” response, resulting in repeated surges of physiological arousal. At the same time, repeated exposure to stress is associated with reduced parasympathetic nervous system (PNS) activity, the system responsible for calming the body and supporting recovery (Winzeler et al., 2017; Waxenbaum et al., 2025). When this parasympathetic “brake” is less effective, individuals experience prolonged physiological arousal and reduced autonomic flexibility (Brosschot et al., 2006).

Critically, these physiological states exert direct effects on neural functioning through bottom-up pathways. Heightened physiological arousal impairs prefrontal cortical activity while amplifying amygdala responsivity, which prioritizes rapid detection of danger over deliberative processing (Arnsten, 2009). Chronic activation of the HPA axis further compounds these effects. Although acute cortisol release is adaptive, repeated or prolonged cortisol exposure disrupts prefrontal regulation, weakens inhibitory control over limbic circuitry, and impairs hippocampal involvement in contextual learning and stress recovery (McEwen, 2007a; Lupien et al., 2009a; McEwen, 2012; Wang et al., 2020). Thus, when prefrontal top-down regulatory processes are disrupted by repeated activation of physiological bottom-up processes, individuals’ capacity to engage in cognitive-affective mindfulness strategies may be particularly difficult or inadvertently amplify distress rather than promote regulation (Lindahl et al., 2017; Canby et al., 2025).

### 2.3 ACEs and lack of biopsychological calibration

Taken together, these cognitive, emotional, and physiological adaptations suggest that difficulties engaging in mindfulness practice among individuals with ACE histories do not reflect a lack of capacity or motivation, but rather a fundamental mismatch between how stress-regulation systems have been calibrated by early adversity and what many standard mindfulness practices involve (Table 1). In a cyclical fashion, repeated exposure to stress leads to consistent activation of bottom-up regulatory systems, which over time, disrupts top-down regulatory systems; in turn, these top-down disruptions constrain control over rapid threat responses and lead to increased activation of bottom-up regulatory systems, thereby further reinforcing the cycle. Therefore, while early adversity tunes regulatory systems toward threat-responsive and defense-oriented states, many cognitive-affective mindfulness practices presume a baseline capacity for bodily safety and awareness, attentional stability, and tolerance of internal experience. When this assumption is violated, practices that rely on inward attention, acceptance, and nonreactivity may feel effortful, destabilizing, or even unsafe, rather than restorative (Lindahl et al., 2017; Canby et al., 2025). This mismatch may help explain mixed findings and limitations observed in MBI research among ACE-affected populations (Spidel et al., 2019; Zhu et al., 2019; Kirlic et al., 2020; Moyes et al., 2022). More so, continuing to rely on traditional mindfulness frameworks is likely to continue to produce inconsistent or insufficient MBIs for individuals whose stress regulation systems were shaped by early adversity.

**Table 1:** Parallel and opposing impacts of adverse childhood experiences (ACEs) and mindfulness-based recalibration on stress-regulatory systems.

Regulatory domain	ACEs	Mindfulness-based recalibration
Overall regulatory orientation	Bias toward threat detection, defense, and survival-oriented responding; stress systems calibrated for rapid danger response	Bias toward safety, flexibility, and recovery; stress systems recalibrated toward adaptive regulation
Top-down prefrontal regulation	Disrupted prefrontal functioning; heightened amygdala reactivity and rapid threat appraisal	Strengthened prefrontal engagement; improved modulation of amygdala reactivity and reduced threat appraisals
Bottom-up physiological regulation	Increased SNS activity; diminished autonomic flexibility; impaired stress responses and recovery	Increased PNS activity; autonomic flexibility; enhanced stress responses and recovery
Directionality of learning	Self-reinforcing cycle of dysregulation: bottom-up hyperarousal disrupts top-down control, which further amplifies arousal	Self-reinforcing cycle of recalibration: bottom-up stabilization enables top-down regulation, which further supports physiological balance
Temporal process	Developmental, experience-dependent calibration toward threat across early life	Experience-dependent recalibration toward safety and flexibility through repeated practice

## 3 The mindfulness recalibration model

**Figure 1:**
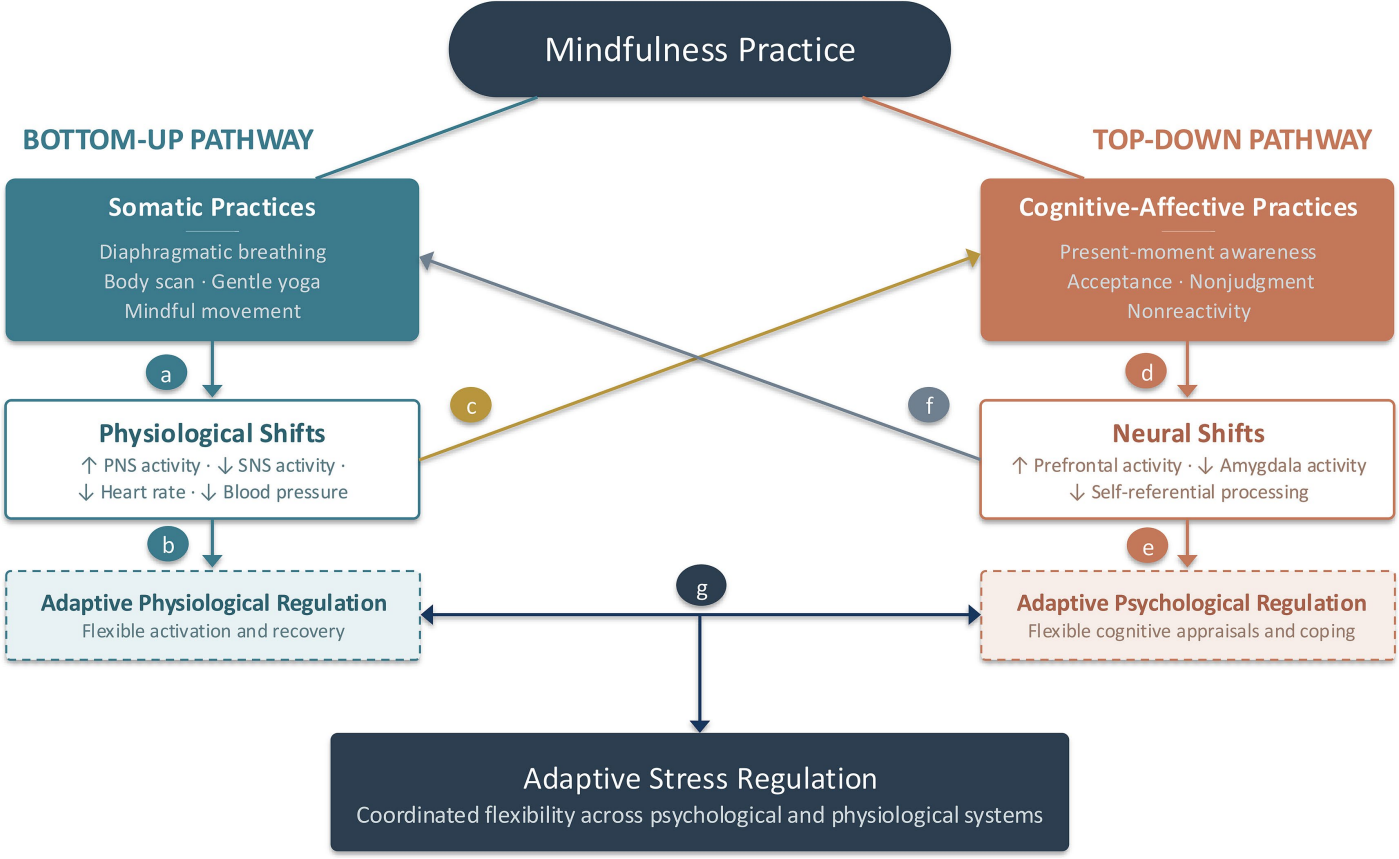
Conceptual model of the mindfulness recalibration model. This figure illustrates the distinct yet complementary roles of somatic and cognitive-affective mindfulness components in recalibrating stress regulation. Somatic practices primarily initiate change via bottom-up pathways by directly downshifting physiological arousal **(a)**. These immediate shifts toward physiological stability provide repeated opportunities for the system to experience successful downregulation, which, over time, strengthens the capacity to flexibly transition between states of reactivity and recovery **(b)**, thereby supporting more adaptive physiological and psychological stress responses **(g)**. Critically for ACE-affected populations, physiological downshifting afforded by somatic mindfulness practices may serve as an essential entry point into effective mindfulness engagement, enabling access to cognitive-affective mindfulness practices that may otherwise be difficult to engage under conditions of hyperarousal **(c)**. Building upon this foundation, cognitive-affective mindfulness practices primarily operate through top-down pathways by recruiting prefrontal activity and dampening amygdala activity **(d)**, thereby enhancing psychological capacities **(e)** used to modulate downstream affective and physiological responses to stress **(g)**. Additionally, these neural shifts may further reinforce somatic practices by enhancing sustained, nonjudgmental attention to bodily cues and reducing re-escalation into threat states **(f)**. Together, these processes promote and sustain adaptive mind–body stress regulation by cultivating dynamic flexibility across psychological and physiological systems **(g)**.

The MRM reframes the central aim of mindfulness interventions as a process of mindfulness-based recalibration that unfolds through practice itself (Table 2). From this perspective, mindfulness practice is understood not solely as the uniform acquisition of cognitive-affective skills, but as an experience-dependent process through which bottom-up physiological and top-down cognitive-affective regulatory systems are progressively reshaped through the coordinated engagement of somatic and cognitive-affective mindfulness practices (Figure 1). By targeting these systems through distinct and complementary processes, mindfulness interventions are conceptualized as facilitating durable shifts in stress-regulatory functioning that support adaptive stress regulation. Adaptive stress regulation in this context refers regulatory flexibility, or the capacity to mount proportionate physiological and psychological responses to stressors and to recover efficiently, rather than remaining locked into patterns of chronic activation or suppression shaped by chronic stress and trauma.

**Table 2:** Traditional framing compared with mindfulness recalibration model (MRM) framing of mindfulness-based interventions (MBIs) for ACE-affected populations.

Conceptual dimension	Traditional framing	MRM framing
Primary target of intervention	Cultivation of mindfulness as a cognitive-affective construct	Recalibration of dysregulated stress systems through coordinated mind–body regulation
Role of somatic practices	Secondary, introductory, or supportive components	Key mechanism of change, particularly for ACE-affected populations
Role of cognitive-affective mindfulness practices	Primary drivers of intervention effects (e.g., awareness, nonjudgment)	Complementary and reinforcing processes that build upon physiological stabilization
Directionality of change	Top-down neural modulation of peripheral physiology	Reciprocal peripheral–central integration
Temporal process	Gradual strengthening of trait mindfulness through repeated cognitive-affective practice	Iterative, experience-dependent regulatory learning that progressively restores bottom-up and top-down flexibility
Implications for intervention design	Uniform protocols emphasizing cultivating ‘mindfulness’	Tailored interventions prioritizing somatic practices and staged integration of cognitive-affective practices
Primary intervention evaluation	Pre-post assessment of selected outcomes	Multi-system assessment of within-practice processes, state-level regulatory changes across the intervention, and downstream outcomes at post-intervention and follow-up

As such, this perspective is especially relevant for individuals with stress- and adversity-shaped regulatory systems, particularly those with histories of ACEs. At the same time, it is important to note that the types of stress-regulatory disruptions central to this framework are not unique to early life adversity; similar patterns have been documented among adults exposed to trauma later in life, including combat veterans and survivors of interpersonal violence (Pole, 2007; Pitman et al., 2012). Accordingly, the MRM may have broader relevance across trauma-exposed populations (See Section 4.3). However, we focus specifically on ACE-affected individuals for several reasons. Early adversity unfolds during sensitive developmental periods when stress-regulatory systems are still maturing, which may result in more deeply entrenched and developmentally embedded patterns of dysregulation compared to trauma experienced later in life (Lupien et al., 2009b). ACEs are also a well-characterized and extensively studied exposure, which provides a strong empirical foundation for developing and testing mechanistic models that can later be extended to other populations. In addition, ACEs are highly prevalent, far exceeding rates of diagnosable PTSD, which makes them especially relevant for understanding population-level risk and identifying modifiable pathways with broad public health impact (Swedo, 2023).

As described above, ACE-related disruptions may limit the effectiveness of mindfulness practices that rely heavily on sustained attention or nonjudgmental awareness (Zhu et al., 2019; Canby et al., 2025). Thus, rather than layering cognitively demanding practices onto already constrained regulatory systems, the MRM proposes that mindfulness interventions may be most effective for ACE-affected populations when they directly engage the same regulatory systems altered by early adversity, but in an adaptive direction. Maladaptive stress regulation is proposed to be an entrenched pattern shaped by adaptation to unsafe or unpredictable environments (McEwen, 2007b; Danese and McEwen, 2012). Thus, within the MRM, mindfulness practice functions as a series of repeated experiences in which individuals learn, both physiologically and psychologically, that they are safe. The goal of mindfulness, therefore, is to provide opposing experiences that allow the mind and body to gradually relearn and reshape these patterns toward adaptive stress regulation.

Importantly, the MRM does not diminish the therapeutic significance of cultivating a nonjudgmental stance toward one’s experiences. Indeed, reducing self-judgment and normalizing distress responses are widely recognized as essential elements of effective trauma-informed care (Sweeney et al., 2018), and substantial evidence identifies the mitigation of negative trauma-related cognitions as a key mechanism of posttraumatic symptom change (Kangaslampi and Peltonen, 2022; Alpert et al., 2023). Rather, the MRM’s position is that the capacity to sustain nonjudgmental awareness may be functionally constrained, not absent, among individuals whose stress-regulatory systems are calibrated toward chronic threat detection. The MRM treats nonjudgment as an emergent capacity that becomes increasingly accessible as physiological conditions shift to support reflective, rather than reactive, engagement with internal experience.

Therefore, within the MRM, somatic mindfulness practices play a foundational role in this process by directly engaging bottom-up physiological regulatory systems and facilitating a felt sense of stability and safety. These practices support balanced and flexible physiological conditions (Ditto et al., 2006; Peressutti et al., 2011; Kirk and Axelsen, 2020; Natarajan, 2023) in which higher-order cognitive processes can function more effectively (Arnsten, 2009; Thayer et al., 2009). To that end, cognitive mindfulness practices then serve as complementary mechanisms, supporting regulation through top-down pathways. Over time, repeated engagement with both practice types reduces the need for chronic threat anticipation, allowing regulatory systems to recalibrate towards flexible, context-appropriate responding. Thus, by adopting a perspective that explicitly accounts for adversity-shaped regulation, MBIs can be more optimized to precisely align with the needs of ACE-affected populations.

We acknowledge that many of the somatic practices included in MBIs – such as breathing exercises, yoga, and mindful movement—are not exclusively “mindfulness practices” in the traditional psychological sense. However, we view this observation not as a limitation of the present framework, but as highlighting a broader conceptual tension within the mindfulness literature. Namely, attributing outcomes solely to the cultivation of mindfulness may obscure the role of these embedded somatic practices that are consistently included in MBIs. For individuals with histories of ACEs in particular, the effectiveness of such interventions may stem not only from the development of mindfulness as a cognitive-affective capacity, but also from the inclusion of body-based regulatory practices and their coordinated interaction with cognitive processes. In this sense, somatic practices may play a primary, not secondary, role in shaping intervention effects. Disentangling the contributions of these distinct components is therefore essential for clarifying when, why, and for whom MBIs are most effective. Accordingly, the MRM provides a conceptual framework for conceptualizing MBIs as processes that unfold through the distinct yet complementary contributions of somatic and cognitive mindfulness practices.

The following sections elaborate on these components and their roles within the model, including their mechanisms of action and their dynamic interaction across bottom-up and top-down regulatory pathways. However, it is first important to clarify how the terms “bottom-up” and “top-down” are used within the MRM, as these designations carry varying meanings across the emotion regulation and neuroscience literatures. Chiesa et al. (2013) previously examined whether mindfulness functions as a top-down or bottom-up emotion regulation strategy, noting that early-stage mindfulness practice may rely more heavily on top-down attentional control mechanisms, while advanced practice may increasingly engage bottom-up perceptual and interoceptive processes. Within the MRM, we use these terms to distinguish the primary regulatory pathway through which a given practice exerts its initial effects, rather than to imply that each practice type operates through a single, isolated system.

Specifically, we designate somatic mindfulness practices (e.g., diaphragmatic breathing, gentle yoga, mindful movement) as “bottom-up” because their primary point of entry into the regulatory system is through direct engagement of peripheral physiological processes, which then influence central neural functioning via afferent signaling pathways. We designate cognitive-affective mindfulness practices (e.g., nonjudgmental awareness, decentering, attentional monitoring) as “top-down” because their primary point of entry is through neurally mediated processes that modulate peripheral physiological stress responses. However, we acknowledge that most mindfulness techniques recruit both central and peripheral systems to varying degrees and are better understood as falling along a spectrum. Some practices are more explicitly somatic in their entry point, others more explicitly cognitive-affective, while others—such as focused attention to the breath, which involves both direct respiratory-autonomic engagement and attentional deployment of prefrontal networks (Chiesa et al., 2013)—occupy an intermediate position. The MRM’s contribution lies not in claiming that these pathways are fully independent, but in proposing that the relative emphasis on bottom-up versus top-down entry points carries important implications for how mindfulness interventions are designed, sequenced, and delivered to populations with adversity-shaped stress-system dysregulation.

### 3.1 Bottom-up somatic mindfulness practices

A core feature of MRM is the emphasis on somatic mindfulness practices as a central driver of intervention effects, especially for individuals with histories of ACEs. Somatic practices – such as diaphragmatic breathing, gentle yoga, and mindful walking (Houser, 2015; Fields, 2019; Classen et al., 2021) – primarily operate through bottom-up pathways by increasing parasympathetic, or vagal, activity (Wang et al., 2010; Jerath et al., 2012; Brown et al., 2013; Wei et al., 2016). These state-level increases in vagal tone counteracts sympathetic dominance and reduces physiological arousal (Lehrer and Gevirtz, 2014). In this more balanced and stable physiological state, prefrontal networks involved in top-down regulation are better able to engage (Arnsten, 2009). In other words, individuals can remain in contact with internal experiences without triggering further autonomic escalation or avoidance, which creates opportunities for successful downregulation. This is consistent with neurovisceral integration models linking vagal tone to executive function and emotion regulation capacity (Thayer and Lane, 2000; Thayer et al., 2009).

Furthermore, across repeated practice, these transient increases in vagal activity are proposed to strengthen the flexibility of autonomic responding. Acute increases in vagal tone during somatic practices provide repeated opportunities for successful autonomic downregulation by reinforcing the capacity to shift out of heightened arousal states (Porges et al., 1994; Lehrer and Gevirtz, 2014). Over time, these repeated regulatory experiences are thought to enhance the dynamic range of vagal control, which supports more flexible patterns of physiological responding (Kok and Fredrickson, 2010). That is, the system becomes better able to mount appropriate reactivity to challenge and to efficiently recover following stressor offset (Waxenbaum et al., 2025). In this way, short-term increases in vagal tone during somatic practices may function as repeated “training signals” that reinforce flexible autonomic regulation over time. Accordingly, MBIs designed to incorporate explicitly somatic practices and directly target bottom-up physiological regulation may be particularly well suited for individuals with histories of ACEs.

Empirical work with ACE-affected populations highlights the importance of these bottom-up, body-focused components. For instance, in a recent review evaluating MBIs for individuals with early adversity (Moyes et al., 2022), the interventions with an emphasis on somatic practices demonstrated significant benefits across multiple domains. Yoga-based interventions (Houser, 2015; Fields, 2019) showed reduced psychological and physiological symptoms, increased mindfulness, enhanced body reconnection, and improved emotion regulation. A body-oriented intervention focused on sensorimotor therapies specifically designed to address chronic threat states showed improvements in anxiety, body awareness, and soothing receptivity, with effects maintained at six-month follow-up (Classen et al., 2021). Although encouraging, the efficacy of these interventions also varied across study settings and outcomes. One of the yoga-based interventions showed significant improvements at only one of the intervention sites (Houser, 2015). Additionally, the body-oriented intervention had no significant impact on depression or PTSD (Classen et al., 2021). Together, these mixed findings suggest that while somatic mindfulness practices can confer meaningful benefits for ACE-affected populations, somatic practices alone may be insufficient.

### 3.2 Top-down cognitive-affective mindfulness practices

The MRM further conceptualizes cognitive-affective practices as complementary and reinforcing elements of mindfulness-based recalibration. These practices, which are consistently defined and implemented within MBIs, typically involve ‘cultivating mindfulness’ through sustained, nonjudgmental awareness of present-moment experiences (Zhang et al., 2021a). This state of consciousness is thought to promote decentering, or seeing internal events as transient mental phenomena rather than accurate reflections of the self (Shapiro et al., 2006). This allows individuals to view situations through a more objective and clear perspective, rather than a stress-reactive one, which in turn creates the opportunity to reappraise situations and adopt more adaptive coping strategies (Garland et al., 2015). In line with cognitive theories of stress and emotion, this shift in cognitive appraisal is proposed to shift downstream physiological responses by reducing threat-related activation and facilitating more adaptive patterns of autonomic and neuroendocrine regulation (Lazarus, 1991; Blascovich and Tomaka, 1996; Jamieson et al., 2012). Stress buffering accounts of mindfulness (Creswell and Lindsay, 2014) describe this top-down pathway through a biological lens, in which engagement with these practices recruits prefrontal activity (e.g., PFC) and dampens limbic activity (e.g., amygdala), thereby modulating downstream physiological responses (Allen et al., 2012; Taren et al., 2015).

However, as discussed above, the very top-down regulatory capacities that support engagement with such practices are often compromised among individuals with histories of ACEs, which may paradoxically limit accessibility to these practices when introduced in isolation (Lindahl et al., 2017; Canby et al., 2025). For example, prior work examining Acceptance and Commitment Therapy (ACT), which is a cognitively oriented intervention focused on fostering psychological flexibility through acceptance of difficult internal experiences and commitment to values-driven behavior, has produced mixed findings among ACE-affected populations (Spidel et al., 2018, 2019). Moreover, even among non-ACE populations, evidence for top-down effects of mindfulness practices remains inconsistent. For example, Baumgartner and Schneider (2023) found that an MBSR intervention did not significantly alter physiological stress reactivity despite improvements in psychological appraisals. This highlights a potential disconnect between top-down cognitive-affective changes and bottom-up physiological regulation, further suggesting the need to target these respective pathways through an emphasis on both cognitive-affective and somatic practices.

### 3.3 Dynamic and reciprocal processes of mindfulness practices

The MRM posits that effective mindfulness training, particularly for ACE-affected populations, depends on the coordinated engagement of somatic and cognitive-affective mindfulness practices, which operate through complementary bottom-up and top-down regulatory pathways. Accordingly, mindfulness-based recalibration is optimized when both pathways are engaged in concert rather than when either component is implemented in isolation.

Importantly, the relationship between somatic and cognitive-affective practices is bidirectional. The MRM emphasizes somatic mindfulness practices as a critical entry point for ACE-affected populations. By directly downshifting physiological arousal (Peressutti et al., 2011; Cruess et al., 2015; Durocher et al., 2018; Kirk and Axelsen, 2020; Natarajan, 2023), somatic practices can begin dismantling entrenched patterns of arousal and threat-related activation. This shift toward physiological balance may temporarily relieve constraints on prefrontal functioning imposed by chronic stress (Arnsten, 2009; Thayer et al., 2009), thereby enabling engagement with cognitive-affective mindfulness practices that may otherwise be difficult to access. Additionally, as cognitive-affective mindfulness skills such as attentional stability and nonjudgment develop, they may enhance the effectiveness of somatic practices by supporting sustained interoceptive engagement and acceptance of bodily sensations, thereby allowing physiological downshifting to occur more efficiently and reliably. This integration may also be especially beneficial for individuals with histories of ACEs, for whom chronic stress exposure can foster patterns of mind–body disconnection or disengagement from internal bodily experience (Schmitz et al., 2023). Taken together, within the MRM, both types of practices are not merely complementary in their downstream effects, but mutually enabling in their implementation.

#### 3.3.1 Evidence from mindfulness-based stress reduction (MBSR)

From this perspective, MBIs that intentionally engage and tailor both bottom-up and top-down regulatory processes in a particular manner may be particularly beneficial for ACE-affected individuals. To illustrate this point, we draw on mindfulness-based stress reduction (MBSR), one of the most widely implemented standardized eight-week mindfulness programs. MBSR systematically incorporates both somatic practices—including body scans, gentle yoga, and breathing exercises – and cognitive-affective practices that cultivate sustained, nonjudgmental awareness of internal and external experiences (de Vibe et al., 2012; Kabat-Zinn, 2003). While MBSR conceptually aligns with the MRM feature of including both typers of practices, null findings from prior studies suggest that standard MBSR may be insufficient for ACE-affected individuals without specifically tailored sequencing and emphasis of intervention elements (Jee et al., 2015).

Alternatively, in a trauma-informed adaptation of MBSR, Kimbrough et al. (2010) incorporated modifications designed to enhance safety and accessibility, including slower pacing, explicit emphasis on choice and agency, normalization of trauma-related responses, and careful titration of inward-focused attention. These adaptations may have reduced the likelihood of overwhelming physiological activation and allowed participants to engage more fully with both somatic and cognitive components of the program. As such, findings included improvements in mindfulness and reductions in depression, anxiety, and PTSD symptoms, which were maintained at follow-up. Comparing results from the unmodified and trauma-informed MBSR studies further illustrate a central premise of the MRM; that the effectiveness of MBIs for ACE-affected populations depends not only on the inclusion and integration of somatic and cognitive-affective practices, but on how they are sequenced, emphasized, and delivered to engage bottom-up and top-down pathways in a mutually reinforcing manner.

### 3.4 Iterative and self-reinforcing progression of recalibration

**Figure 2:**
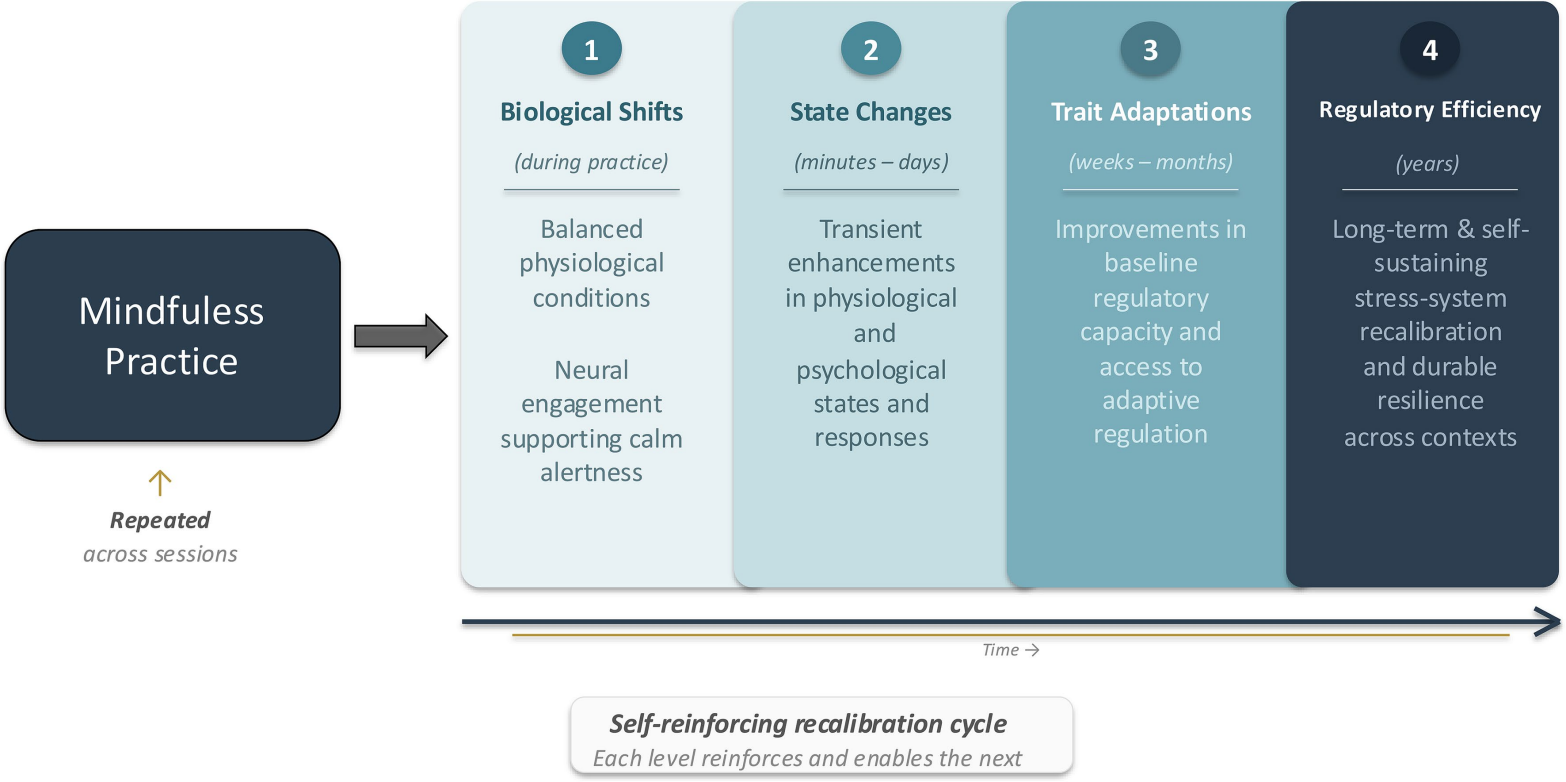
Temporal progression of mindfulness-based recalibration. This figure depicts how coordinated engagement of somatic and cognitive-affective mindfulness practices establish the foundational conditions for regulatory change across nested temporal levels. At Level 1, within-session somatic and cognitive-affective practices elicit immediate autonomic and neural shifts that establish balanced physiological conditions and calm alertness. For ACE-affected individuals, extended engagement with somatic practices may be particularly critical at this stage, as physiological downshifting can facilitate access to cognitive-affective practices and initiate regulatory learning. At Level 2, these biological shifts manifest as transient improvements in psychological and physiological states. With practice repetition and application in daily life, state-level changes consolidate into Level 3 trait-like adaptations, reflected in improved baseline regulatory capacity and access to adaptive regulation across contexts. Over time, Level 4 reflects the emergence of increasingly efficient, self-sustaining regulatory patterns, as repeated instances of psychological and physiological flexibility reinforce adaptive stress regulation, culminating in durable stress-system recalibration and resilience.

To clarify how these practices work together, which elements drive early-stage change, and how these mechanisms translate into longer-term benefits among ACE-affected populations, the MRM proposes that mindfulness-based recalibration operates through an iterative and self-reinforcing cycle. Broadly, the psychological and physiological adaptations conferred through mindfulness practice mutually reinforce one another, recalibrating the system toward adaptive modes of stress regulation. Adaptive stress regulation itself across daily life contexts further reinforces physiological and cognitive-affective flexibility (McEwen, 2007b; Thayer et al., 2009), such that these practices and regulatory responses are increasingly learned, automatized, and enacted with minimal effort over time. In this way, the benefits of mindfulness practices emerge through the same iterative learning dynamics by which early adversity shapes maladaptive stress regulation, but operating in an adaptive direction through repeated, structured exposure to regulated and balanced mind–body states, rather than the chronic, threat-driven states observed during stress and traumatic experiences. The following sections further clarifies this process by proposing the distinct temporal levels through which mindfulness-based recalibration may work (Figure 2). In the sections that follow, we organize existing empirical findings across four temporal levels – immediate biological shifts, short-term state changes, longer-term trait adaptations, and enduring regulatory efficiency – to illustrate how mindfulness practices may interactively recalibrate dysregulated stress systems across time.

#### 3.4.1 Immediate biological shifts during practice

Within the MRM, immediate shifts in biological systems during mindfulness practices are conceptualized as the first phase of mindfulness-based recalibration. Somatic mindfulness practices, such as mindful breathing and body-focused awareness exercises, have been shown to acutely increase parasympathetic activity and reduce sympathetic arousal, producing immediate shifts toward physiological regulation (Peressutti et al., 2011; Kirk and Axelsen, 2020; Natarajan, 2023). These autonomic changes are often accompanied by reductions in heart rate and blood pressure (Li et al., 2018), which may reflect decreased sympathetic activation, increased parasympathetic engagement, or both. For instance, in some practices, these shifts co-occur with a balanced pattern of calm alertness rather than passive relaxation (Ditto et al., 2006).

During cognitive-affective mindfulness practices, such as nonjudgmental present-moment awareness, neural activity shifts away from habitual self-referential and evaluative processing and toward patterns associated with decentering, attentional stability, and emotion regulation (Fennell et al., 2016; Tang et al., 2015). Consistent findings include reduced activation within midline cortical regions implicated in rumination and self-focus, alongside enhanced engagement of regulatory and attentional networks (Ives-Deliperi et al., 2011; Doll et al., 2016). Electrophysiological evidence further suggests increased flexibility in alpha and theta oscillatory activity, which reflect a neural state that supports sustained attention without heightened arousal (Ahani et al., 2013; Faber et al., 2015; Rodriguez-Larios et al., 2020).

These immediate biological shifts are especially consequential for understanding how mindfulness practice initiates regulatory change, particularly among ACE-affected populations. In the moment, somatic-driven shifts toward physiological stability and balance may be sufficient to temporarily release constraints on prefrontal functioning (Thayer et al., 2009), allowing cognitive mindfulness practices to become accessible immediately following, or even concurrently with, somatic engagement. Consistent with this mechanism, many mindfulness practices begin by orienting attention to the breath or bodily sensations (Kabat-Zinn, 2003). Thus, these practices directly leverage somatic elements to scaffold subsequent cognitive-affective engagement. Importantly, the MRM further establishes that for individuals with histories of ACEs, a single instance of regulated physiological responding may not be sufficient to reliably support sustained cognitive engagement. In these cases, repeated exposure to somatic-driven biological shifts across practice sessions may be necessary to gradually recalibrate basal physiological functioning and reduce chronic threat readiness, while increasing autonomic flexibility over time. As these regulated states become more familiar and accessible through repetition, cognitive mindfulness practices may become progressively less effortful, more stable, and more readily integrated within formal practice.

Furthermore, these within-practice biological shifts are especially important because they highlight the active role of mindfulness practice itself as the primary driver of regulatory change, rather than treating post-intervention outcomes as the starting point of explanation. Although these biological shifts may be transient within any single practice session, each repeated engagement provides the stress-regulatory system with direct experience of operating in a regulated, non-defensive mode. Through repetition, these states become increasingly familiar, accessible, and efficient. Therefore, these practices reflect experience-dependent learning within the same biological systems that are engaged during stress and trauma (May, 2011). Just as repeated exposure to threat-oriented states during adverse experiences shapes maladaptive regulatory patterns (McEwen, 2007b), repeated exposure to regulated and safe internal states during mindfulness practice may progressively reshape these systems in an adaptive direction. This provides the biological foundation upon which state-level, trait-like, and enduring regulatory changes iteratively build within mindfulness-based recalibration.

#### 3.4.2 State-level shifts after short-term practice

Within the MRM, the transition from immediate biological shifts during practice to state-level benefits following short-term practice is conceptualized as an early indicator of mindfulness-based recalibration, rather than as discrete or transient outcomes. Physiologically, single-session practices—particularly those with a strong somatic emphasis, such as body scans and mindful breathing—have been shown to reduce resting heart rate, respiration rate, and cardiovascular load, alongside shifts toward greater autonomic balance (35, 57). Beyond cardiovascular and autonomic shifts, somatic practices appear to modulate cortisol responses during acute stressors early in training relative to cognitive-affective practices (Cruess et al., 2015), as direct bottom-up regulation may be more readily accessible than cognitive, top-down pathways.

Furthermore, just by simply shifting towards a more balanced physiological state, individuals are better positioned to adjust how they perceive and process stressors. In line with this perspective, following a brief mindfulness induction or short-term training, individuals commonly exhibit increases in state mindfulness alongside reductions in negative affect, subjective stress, and anxiety, as well as improvements in attentional stability and executive functioning (Durocher et al., 2018; Schumer et al., 2018; Leyland et al., 2019; Williams et al., 2024; Zainal et al., 2024). Conceptually, the immediate reductions in arousal paired with preserved alertness may facilitate prefrontal activity that better allows for internal and external signals to be attended to in an accepting, non-reactive manner (Arnsten, 2009; Thayer et al., 2009), rather than avoided or amplified by threat-based processing.

This progression underscores the self-reinforcing nature of mindfulness-based recalibration. As immediate shifts during practice give rise to state-level changes in affect, attention, and stress appraisal (Durocher et al., 2018; Schumer et al., 2018; Leyland et al., 2019; Williams et al., 2024; Zainal et al., 2024), subsequent encounters with stress may be processed under less defensive physiological conditions (Brosschot and Thayer, 2003). These state-level experiences provide additional corrective feedback to biological and psychological systems, thereby reinforcing patterns of physiological regulation and cognitive-affective flexibility (May, 2011). Over repeated practice sessions and daily life applications, this reciprocal engagement becomes increasingly learned, such that regulated physiological states more readily support adaptive psychological responses, and adaptive psychological responses further sustain physiological regulation. Moreover, these shifts may further enhance engagement with mindfulness practice itself, making it more accessible and effective. In this way, state-level benefits are active contributors to an iterative cycle through which stress-regulatory systems are progressively recalibrated.

#### 3.4.3 Trait-like changes across long-term practice

From an MRM perspective, trait-level adaptations are conceptualized as the downstream products of iterative, self-reinforcing regulatory learning. Repeated engagement with regulated physiological and psychological states – initially supported by within-practice shifts and progressively reinforced through state-level changes recurring across formal sessions and daily life contexts – begin to shape baseline stress-regulatory systems toward greater flexibility and reduced threat sensitivity (May, 2011). In turn, this shift in baseline functioning makes subsequent engagement with mindfulness practices, and with daily stressors themselves, less effortful and more effective. As such, prior work demonstrates that sustained mindfulness training is associated with increases in trait mindfulness alongside improvements in cognitive and emotional regulation, adaptive cortisol responses and recovery, and autonomic flexibility, as well as broader gains in mental and physical health (O’Leary et al., 2016; Morton et al., 2020; Zhang et al., 2021a; Calderone et al., 2024). Within ACE-affected populations, this literature has similarly emphasized trait-level outcomes, with studies demonstrating reductions in depression, anxiety, and trauma-related symptoms, alongside improvements in coping, mood, psychological flexibility, and overall quality of life (Ortiz and Sibinga, 2017; Kirlic et al., 2020; Moyes et al., 2022).

Again, as regulatory responses become more stable, they continuously feed back into the system and further reinforce psychological and physiological flexibility (Thayer and Lane, 2000; McEwen, 2007b). Thus, within the MRM, trait-level changes reflect not simply the accumulation of cognitive-affective skills, but the consolidation of repeated regulatory successes into more stable, enduring patterns of self-reinforcing adaptive stress regulation. For instance, emerging work has found reductions in rumination and self-judgment, alongside improvements in emotion regulation and prefrontal-limbic coordination among ACE-affected individuals following standardized MBIs (Joss et al., 2024; Joss, 2025). Beyond reflecting trait-level benefits, these adaptations also actively support continued regulation by reducing the likelihood of reactivating maladaptive cognitive-affective patterns and physiological reactivity. Moreover, emerging findings suggest potential normalization of stress-related biomarkers, including reduced anticipatory cortisol responses, following MBI participation among ACE-affected populations (Cohen et al., 2021). Such biological adaptations allow for adaptive responses to become increasingly enacted across a wider range of real-world stressors, and further shift regulatory patterns towards adaptive modes of functioning (O’Leary et al., 2016).

#### 3.4.4 Recalibration and emergence of enduring adaptive patterns

As mindfulness-based recalibration becomes increasingly learned and self-sustained through experience-dependent reinforcement over time (May, 2011), the psychological and physiological adaptations are proposed to consolidate into increasingly automatic and embedded regulatory patterns. Although limited, prior work has demonstrated promise in sustained benefits among ACE-affected populations; adults with a history of childhood sexual abuse showed significant long-term improvements in depression, PTSD, anxiety symptoms, and mindfulness scores 2.5 years post-MBSR (Earley et al., 2014). Within the MRM, this long-term phase reflects successful recalibration of ACE-disrupted regulatory systems across domains. Outcomes are theorized to resemble those observed in non-ACE populations or those with naturally high levels of trait mindfulness, who may trend toward having automated an accepting, nonreactive stance toward experiences (Tang et al., 2015). Whereas traditional conceptualizations of MBIs often treat these enduring patterns as the primary goal and mechanism of intervention effects, a core feature of the MRM is the proposition that MBIs should not be designed solely to *directly* cultivate an accepting, nonreactive stance toward experience. Rather, the MRM emphasizes that such qualities are emergent outcomes of a sequence of interrelated practices and regulatory processes. In this view, acceptance and nonreactivity arise gradually through repeated within-practice physiological and psychological shifts that consolidate over time, which are critically shaped by the timing, sequencing, and integration of bottom-up somatic and top-down cognitive-affective mindfulness practices.

In this way, successful stress regulation itself becomes the primary mechanism through which long-term recalibration is progressively learned, stabilized, and maintained across contexts. For individuals with a history of ACEs, the emergence of such regulatory efficiency may be especially consequential, as it directly targets the chronic physiological activation and dysregulated stress responses that contribute to elevated risk for stress-related and inflammatory disease (Danese and McEwen, 2012; Creswell and Lindsay, 2014). From a translational perspective, and in line with previous conceptualizations of MBIs, mindfulness practice is not solely a tool to be applied to discrete problems, but can foster a foundational regulatory capacity that supports resilience and health across contexts (Earley et al., 2014).

## 4 Discussion

### 4.1 Summary

The MRM integrates bottom-up and top-down perspectives on stress regulation to clarify how mindfulness practice may be optimally structured for individuals with histories of early adversity. In doing so, the model also addresses a broader conceptual tension within the mindfulness literature; although many MBIs incorporate somatic elements, their effects are often attributed broadly to the cultivation of mindfulness as a cognitive-affective capacity. The MRM, therefore, provides a framework for disentangling how different practice components may engage distinct regulatory pathways, and how their coordinated engagement may drive intervention effects, particularly among ACE-affected populations. Specifically, the MRM positions somatic mindfulness practices as primary initiators of regulatory change by directly engaging bottom-up pathways, while emphasizing the complementary role of cognitive-affective mindfulness practices in extending and consolidating these gains over time through top-down processes. The model further highlights how practice-level regulatory state shifts, can, through repeated practice and experience-dependent learning, consolidate into more enduring, trait-like regulatory capacities that support long-term biopsychological recalibration.

Within this framework, mindfulness-based recalibration functions as a counter-process to the effects of ACEs. Whereas early adversity and chronic stress bias regulatory systems toward persistent bottom-up threat activation that progressively constrains prefrontal, top-down control and vice versa (Thayer and Lane, 2000; McEwen, 2007b; Arnsten, 2009), mindfulness practice shapes these same systems in the opposite direction; first restoring physiological safety and flexibility, and subsequently re-enabling cognitive-affective regulatory capacities. Through repeated engagement of this adaptive sequence, mindfulness practice has the potential to recalibrate biopsychological systems previously shaped by early adversity, thereby supporting adaptive stress regulation among ACE-affected populations.

### 4.2 Next, steps for empirical research

Although grounded in prior theory and evidence, the MRM requires systematic empirical testing to establish its explanatory and translational relevance for ACE-affected populations. According to the MRM, mindfulness interventions should prioritize somatic practices early and progressively integrate cognitive-affective practices for these populations. As the field currently stands, there is a wide variety of practices included under the MBI “umbrella.” For example, the trauma-informed adaptation of MBSR previously described incorporated weekly sessions that integrated a range of practices – including sitting meditation, body scans, and gentle yoga – alongside explicit instruction in mindful, nonjudgmental awareness applied across both formal practice and everyday life contexts (Kimbrough et al., 2010). As Kirlic et al. (2020) notes, additional research is needed to “identify the most impactful aspects of mindfulness to employ in those with early life stress.”

Accordingly, future studies should directly compare interventions that vary in the relative emphasis and sequencing of somatic and cognitive-affective mindfulness practices to determine how their coordinated engagement supports mindfulness-based recalibration. For example, intervention protocols could be designed to (a) deliver somatic practices exclusively for an initial period before introducing cognitive practices, (b) deliver somatic and cognitive practices in parallel but at distinct points within sessions, or (c) integrate somatic and cognitive practices simultaneously within sessions. In addition, future work should examine variation within somatic practices themselves, which differ substantially across MBIs. Whereas cognitive practices tend to be relatively standardized (e.g., nonjudgmental awareness of breath, thoughts, or emotions), somatic practices range from breathing exercises and body scans to mindful movement, yoga, and walking meditation (de Vibe et al., 2012; Santorelli et al., 2017). Given the critical role of somatic practices within the MRM, systematic comparison of these practices is needed to clarify which types of somatic practices most effectively initiate and sustain recalibration.

Because the MRM posits that mindfulness-based recalibration emerges through iterative, self-reinforcing regulatory cycles, evaluating outcomes at a single time point or relying solely on pre-post change is insufficient to test this core claim. Moreover, given the emphasis on reciprocal psychological and physiological changes unfolding through top-down and bottom-up pathways across time, it is important to employ longitudinal, multi-timepoint designs with biopsychological assessments that can capture dynamic change processes and temporal coupling across systems.

Finally, given the translational utility of mindfulness practices for ACE-affected populations as a means of targeting core disruptions in stress regulation, future research should also evaluate whether mindfulness-based recalibration produces downstream benefits for clinically relevant outcomes. This may include evaluating changes in anxiety, depression, trauma-related symptoms, substance use, eating behaviors, sleep, and other stress-related health behaviors. At the physical health level, translational outcomes may include changes in cardiometabolic risk, inflammatory markers, pain, fatigue, and stress-related disease vulnerability. Although prior work has examined whether improvements in underlying stress-regulatory mechanisms mediate such applied health outcomes among primarily non-ACE populations following standard MBIs (Creswell and Lindsay, 2014), it is necessary to identify whether mindfulness-based recalibration approaches are scalable, sustainable, and capable of supporting long-term health and well-being among individuals exposed to chronic stress or early-life adversity.

### 4.3 Limitations, scope, and future directions

Three primary limitations of the MRM warrant discussion, both to clarify its intended scope and to identify directions in which it may be extended or refined. First, while the MRM is developed with specific reference to ACE-affected populations, the regulatory disruptions it addresses are also observed across a broader range of trauma-exposed groups (Pole, 2007; Pitman et al., 2012). While the developmental entrenchment of stress-regulatory patterns following early adversity provides a particularly compelling case for the sequenced, recalibration-focused approach proposed here, future work should evaluate whether and how the MRM’s principles generalize to populations whose regulatory disruptions were acquired outside of sensitive developmental windows.

Second, the present framework is situated primarily within the mindfulness literature, and does not engage extensively with the broader landscape of evidence-based trauma treatments. This is an intentional scope decision; the MRM is designed to address specific questions within the MBI literature—namely, why MBIs may produce inconsistent or adverse outcomes when applied to ACE-affected populations and what are the mechanisms they operate through—rather than to advance a new model of trauma treatment more broadly. With that being said, established trauma-focused interventions—including those incorporating trauma processing, imagery exposure, cognitive restructuring, and psychoeducation—have demonstrated robust efficacy for posttraumatic stress symptoms, and systematic reviews indicate that multicomponent approaches combining these elements tend to produce the strongest outcomes (Schnyder et al., 2015; Coventry et al., 2020). Thus, the MRM is not intended to supplant these models, nor to suggest that mindfulness-based approaches alone constitute sufficient treatment for trauma-related pathology. Rather, the model addresses how mindfulness practices can be optimally structured to engage the stress-regulatory systems most directly disrupted by early life adversity. In this sense, the MRM may be best understood as a mechanistic framework for one component within a broader, integrative approach to trauma recovery.

For example, the trauma-informed adaptation of MBSR described by Kimbrough et al. (2010) incorporated not only somatic and cognitive-affective mindfulness practices but also explicit psychoeducation, normalization of trauma responses, and careful titration of inward-focused attention. This is consistent with the possibility that mindfulness-based recalibration may function most effectively when embedded within a broader therapeutic framework that includes direct engagement with traumatic experience (Bernstein et al., 2015). Conceptually, the MRM’s temporal progression parallels phase-based approaches to the treatment of complex PTSD, which similarly advocate for physiological safety and balance before trauma processing (de Boer et al., 2021). The MRM extends this logic by specifying the biopsychological mechanisms through which particular mindfulness practices may support each phase, but does not claim that mindfulness practice alone constitutes comprehensive trauma treatment.

Third, the MRM is grounded in a characterization of ACE-related stress-system dysregulation that, while broadly supported, is necessarily simplified. For example, while we emphasize general patterns of heightened sympathetic and reduced parasympathetic activity, the relationship between these autonomic branches is not strictly reciprocal; the SNS and PNS can co-activate, co-inhibit, or change independently depending on the context (Berntson et al., 1991). Similarly, the HPA axis consequences of ACEs may not always be uniform. Some individuals exhibit exaggerated cortisol reactivity while others show blunted responses (al’Absi et al., 2021; Brindle et al., 2022). Adding further complexity, glucocorticoid effects on the brain are region- and timing-dependent, thereby differentially affecting the hippocampus, amygdala, and prefrontal cortex depending on which structures are undergoing critical development at the time of stress exposure (Lupien et al., 2009b). A more detailed examination of these nuanced dynamics is beyond the scope of this paper but represents an important direction for future development of the framework.

### 4.4 Conclusion

By shifting the focus of MBIs from the cultivation of mindfulness itself to the process of stress-system recalibration for ACE-affected populations, the MRM reframes MBIs as experience-dependent processes that are uniquely suited to target the core regulatory disruptions shaped by early adversity. Thus, this framework offers a roadmap for designing interventions that are more developmentally informed and responsive to individual differences in regulatory capacity, particularly through the intentional integration and sequencing of bottom-up somatic and top-down cognitive-affective practices. As such, the MRM highlights the importance of supporting physiological adaptations before broader cognitive-affective engagement, thereby increasing accessibility, effectiveness, and durability of intervention effects. This framework further emphasizes the importance of measuring both psychological and physiological changes across time to inform mechanistically precise interventions that are tailored to the needs of ACE-affected populations. Collectively, this framework advances the field toward a mindfulness-based recalibration approach capable of supporting long-term health, resilience, and well-being among individuals exposed to early-life adversity.

## Data Availability

The original contributions presented in the study are included in the article/supplementary material, further inquiries can be directed to the corresponding author/s.

## References

[ref1] Ahani A. Wahbeh H. Miller M. Nezamfar H. Erdogmus D. Oken B. . (2013). Change in physiological signals during mindfulness meditation. 2013 6th International IEEE/EMBS Conference on Neural Engineering (NER), 1378–1381. doi: 10.1109/NER.2013.6696199 PMC3988787 24748422

[ref2] al’Absi M. Ginty A. T. Lovallo W. R. (2021). Neurobiological mechanisms of early life adversity, blunted stress reactivity and risk for addiction. Neuropharmacology 188:108519. doi: 10.1016/j.neuropharm.2021.108519, 33711348 PMC9195251

[ref3] Allen M. Dietz M. Blair K. S. van Beek M. Rees G. Vestergaard-Poulsen P. . (2012). Cognitive-affective neural plasticity following active-controlled mindfulness intervention. J. Neurosci. 32, 15601–15610. doi: 10.1523/JNEUROSCI.2957-12.2012, 23115195 PMC4569704

[ref4] Alpert E. Shotwell Tabke C. Cole T. A. Lee D. J. Sloan D. M. (2023). A systematic review of literature examining mediators and mechanisms of change in empirically supported treatments for posttraumatic stress disorder. Clin. Psychol. Rev. 103:102300. doi: 10.1016/j.cpr.2023.102300, 37320986

[ref5] Arnsten A. F. T. (2009). Stress signalling pathways that impair prefrontal cortex structure and function. Nat. Rev. Neurosci. 10, 410–422. doi: 10.1038/nrn2648, 19455173 PMC2907136

[ref6] Baer R. Crane C. Miller E. Kuyken W. (2019). Doing no harm in mindfulness-based programs: conceptual issues and empirical findings. Clin. Psychol. Rev. 71, 101–114. doi: 10.1016/j.cpr.2019.01.001, 30638824 PMC6575147

[ref7] Banerjee M. Cavanagh K. Strauss C. (2018). Barriers to mindfulness: a path analytic model exploring the role of rumination and worry in predicting psychological and physical engagement in an online mindfulness-based intervention. Mindfulness 9, 980–992. doi: 10.1007/s12671-017-0837-4, 29875884 PMC5968050

[ref8] Baumgartner J. N. Schneider T. R. (2023). Acute biopsychosocial stress responses in mindfulness meditators and non-meditators: the mediating role of closeness. Mindfulness 14, 1435–1445. doi: 10.1007/s12671-023-02146-z

[ref9] Berntson G.G. Cacioppo J.T. Quigley K.S. (1991). “Autonomic Determinism: The Modes of Autonomic Control, the Doctrineof Autonomic Space, and the Laws of AutonomicConstraint.” Psychological review, 98:459. 10.1037/0033-295x.98.4.459 1660159

[ref10] Blascovich J. Tomaka J. (1996). “The biopsychosocial model of arousal regulation,” in Advances in Experimental Social Psychology, ed. Zanna M. P. (San Diego, CA: Academic Press), 1–51.

[ref11] Brindle R. C. Pearson A. Ginty A. T. (2022). Adverse childhood experiences (ACEs) relate to blunted cardiovascular and cortisol reactivity to acute laboratory stress: a systematic review and meta-analysis. Neurosci. Biobehav. Rev. 134:104530. doi: 10.1016/j.neubiorev.2022.104530, 35031343

[ref12] Brosschot J. F. Gerin W. Thayer J. F. (2006). The perseverative cognition hypothesis: a review of worry, prolonged stress-related physiological activation, and health. J. Psychosom. Res. 60, 113–124. doi: 10.1016/j.jpsychores.2005.06.074, 16439263

[ref13] Brosschot J. F. Thayer J. F. (2003). Heart rate response is longer after negative emotions than after positive emotions. Int. J. Psychophysiol. 50, 181–187. doi: 10.1016/S0167-8760(03)00146-6, 14585487

[ref14] Brown R. P. Gerbarg P. L. Muench F. (2013). Breathing practices for treatment of psychiatric and stress-related medical conditions. Psychiatr. Clin. 36, 121–140. doi: 10.1016/j.psc.2013.01.001, 23538082

[ref15] Brown K. W. Ryan R. M. (2003). The benefits of being present: mindfulness and its role in psychological well-being. J. Pers. Soc. Psychol. 84, 822–848. doi: 10.1037/0022-3514.84.4.822, 12703651

[ref16] Brown K. W. Ryan R. M. Creswell J. D. (2007). Mindfulness: theoretical foundations and evidence for its salutary effects. Psychol. Inq. 18, 211–237. doi: 10.1080/10478400701598298

[ref17] Bernstein A. Hadash Y. Lichtash Y. Tanay G. Shepherd K. Fresco D. M. (2015). Decentering and Related Constructs: A Critical Review and Metacognitive Processes Model. Perspectives on Psychological Science, 10, 599–617. doi: 10.1177/1745691615594577, 26385999 PMC5103165

[ref18] Calderone A. Latella D. Impellizzeri F. de Pasquale P. Famà F. Quartarone A. . (2024). Neurobiological changes induced by mindfulness and meditation: a systematic review. Biomedicine 12:2613. doi: 10.3390/biomedicines12112613, 39595177 PMC11591838

[ref19] Canby N. K. Cosby E. A. Palitsky R. Kaplan D. M. Lee J. Mahdavi G. . (2025). Childhood trauma and subclinical PTSD symptoms predict adverse effects and worse outcomes across two mindfulness-based programs for active depression. PLoS One 20:e0318499. doi: 10.1371/journal.pone.0318499, 39883728 PMC11781677

[ref20] Chiesa A. Serretti A. Jakobsen J. C. (2013). Mindfulness: top–down or bottom–up emotion regulation strategy? Clin. Psychol. Rev. 33, 82–96. doi: 10.1016/j.cpr.2012.10.006 23142788

[ref21] Classen C. C. Hughes L. Clark C. Hill Mohammed B. Woods P. Beckett B. (2021). A pilot RCT of a body-oriented group therapy for complex trauma survivors: an adaptation of sensorimotor psychotherapy. J Trauma Dissociation 22, 52–68. doi: 10.1080/15299732.2020.1760173, 32419670

[ref22] Cohen Z. P. Cosgrove K. T. Akeman E. Coffey S. Teague K. Hays-Grudo J. . (2021). The effect of a mindfulness-based stress intervention on neurobiological and symptom measures in adolescents with early life stress: a randomized feasibility study. BMC Complementary Med. Ther. 21:123. doi: 10.1186/s12906-021-03295-1, 33858395 PMC8050904

[ref23] Coventry P. A. Meader N. Melton H. Temple M. Dale H. Wright K. . (2020). Psychological and pharmacological interventions for posttraumatic stress disorder and comorbid mental health problems following complex traumatic events: systematic review and component network meta-analysis. PLoS Med. 17:e1003262. doi: 10.1371/journal.pmed.1003262, 32813696 PMC7446790

[ref24] Creswell J. D. Lindsay E. K. (2014). How does mindfulness training affect health? A mindfulness stress buffering account. Curr. Dir. Psychol. Sci. 23, 401–407. doi: 10.1177/0963721414547415

[ref25] Cruess D. G. Finitsis D. J. Smith A.-L. Goshe B. M. Burnham K. Burbridge C. . (2015). Brief stress management reduces acute distress and buffers physiological response to a social stress test. Int. J. Stress. Manag. 22, 270–286. doi: 10.1037/a0039130

[ref26] Danese A. McEwen B. S. (2012). Adverse childhood experiences, allostasis, allostatic load, and age-related disease. Physiol. Behav. 106, 29–39. doi: 10.1016/j.physbeh.2011.08.019, 21888923

[ref27] de Boer K. Gnatt I. Mackelprang J. L. Williamson D. Eckel D. Nedeljkovic M. (2021). Phase-based approaches for treating complex trauma: a critical evaluation and case for implementation in the Australian context. Aust. Psychol. 56, 437–445. doi: 10.1080/00050067.2021.1968274

[ref28] de Vibe M. Bjørndal A. Tipton E. Hammerstrøm K. Kowalski K. (2012). Mindfulness based stress reduction (MBSR) for improving health, quality of life, and social functioning in adults. Campbell Syst. Rev. 8, 1–127. doi: 10.4073/csr.2012.3

[ref29] Ditto B. Eclache M. Goldman N. (2006). Short-term autonomic and cardiovascular effects of mindfulness body scan meditation. Ann. Behav. Med. 32, 227–234. doi: 10.1207/s15324796abm3203_9, 17107296

[ref30] Doll A. Hölzel B. K. Mulej Bratec S. Boucard C. C. Xie X. Wohlschläger A. M. . (2016). Mindful attention to breath regulates emotions via increased amygdala–prefrontal cortex connectivity. NeuroImage 134, 305–313. doi: 10.1016/j.neuroimage.2016.03.041, 27033686

[ref31] Durocher J. J. Marti H. Morin B. Wakeham T. R. (2018). Single session mindfulness meditation reduces aortic pulsatile load and anxiety in mild to moderately anxious adults. FASEB J. 32, 714.19–714.19. doi: 10.1096/fasebj.2018.32.1_supplement.714.19

[ref32] Earley M. D. Chesney M. A. Frye J. Greene P. A. Berman B. Kimbrough E. (2014). Mindfulness intervention for child abuse survivors: a 2.5-year follow-up. J. Clin. Psychol. 70, 933–941. doi: 10.1002/jclp.22102 24844944

[ref33] Faber P. L. Lehmann D. Gianotti L. R. R. Milz P. Pascual-Marqui R. D. Held M. . (2015). Zazen meditation and no-task resting EEG compared with LORETA intracortical source localization. Cogn. Process. 16, 87–96. doi: 10.1007/s10339-014-0637-x, 25284209

[ref9001] Fareri D. S. Tottenham N. (2016). Effects of early life stress on amygdala and striatal development. Developmental Cognitive Neuroscience, 19, 233–247. doi: 10.1016/j.dcn.2016.04.005 27174149 PMC4912892

[ref34] Fennell A. B. Benau E. M. Atchley R. A. (2016). A single session of meditation reduces of physiological indices of anger in both experienced and novice meditators. Conscious. Cogn. 40, 54–66. doi: 10.1016/j.concog.2015.12.010, 26748026

[ref35] Fields A. (2019). The Impact of Therapeutic Yoga on Adult Female Survivors of Child Sex Abuse in Nicaragua: Exploring the Mind-Body Relationship in a Culturally Embedded Healing Process. Ph.D. Universidad Centroamericana. Nicaragua. Available online at: https://www.proquest.com/docview/2318681283/abstract/E6F628675E994B66PQ/1 (Accessed December 20, 2025).

[ref36] Garland E. L. Farb N. A. Goldin P. R. Fredrickson B. L. (2015). The mindfulness-to-meaning theory: extensions, applications, and challenges at the attention–appraisal–emotion interface. Psychol. Inq. 26, 377–387. doi: 10.1080/1047840X.2015.1092493

[ref37] Gu J. Strauss C. Bond R. Cavanagh K. (2015). How do mindfulness-based cognitive therapy and mindfulness-based stress reduction improve mental health and wellbeing? A systematic review and meta-analysis of mediation studies. Clin. Psychol. Rev. 37, 1–12. doi: 10.1016/j.cpr.2015.01.006, 25689576

[ref38] Gunduz A. Gundogmus I. Engin B. Işler A. Sertcelik S. Yasar A. (2019). Effects of adverse childhood events over metacognitions, rumination, depression and worry in healthy university students. Ann. Med. Res. 1. doi: 10.5455/annalsmedres.2019.05.269

[ref39] Houser M. (2015). A Mixed Methods Evaluation of the Effectiveness of a Group Yoga Intervention as an Adjunctive Trauma Therapy for Adolescent Girls,” Electronic Theses and Dissertations. Available online at: https://digitalcommons.du.edu/etd/1063

[ref40] Ives-Deliperi V. L. Solms M. Meintjes E. M. (2011). The neural substrates of mindfulness: an fMRI investigation. Soc. Neurosci. 6, 231–242. doi: 10.1080/17470919.2010.513495, 20835972

[ref41] Jamieson J. P. Nock M. K. Mendes W. B. (2012). Mind over matter: reappraising arousal improves cardiovascular and cognitive responses to stress. J. Exp. Psychol. Gen. 141, 417–422. doi: 10.1037/a0025719, 21942377 PMC3410434

[ref42] Jee S. H. Couderc J.-P. Swanson D. Gallegos A. Hilliard C. Blumkin A. . (2015). A pilot randomized trial teaching mindfulness-based stress reduction to traumatized youth in foster care. Complement. Ther. Clin. Pract. 21, 201–209. doi: 10.1016/j.ctcp.2015.06.007 26256140

[ref43] Jerath R. Barnes V. A. Dillard-Wright D. Jerath S. Hamilton B. (2012). Dynamic change of awareness during meditation techniques: neural and physiological correlates. Front. Hum. Neurosci. 6:131. doi: 10.3389/fnhum.2012.00131, 23060766 PMC3443747

[ref44] Joss D. (2025). Mindfulness-based interventions for preventing childhood maltreatment and restoring health among survivors: a public health perspective. Mindfulness 16, 1757–1764. doi: 10.1007/s12671-025-02585-w, 40837239 PMC12364083

[ref45] Joss D. Teicher M. H. Lazar S. W. (2024). Temporal dynamics and long-term effects of a mindfulness-based intervention for young adults with adverse childhood experiences. Mindfulness 15, 2245–2261. doi: 10.1007/s12671-024-02439-x, 40160902 PMC11951444

[ref46] Kabat-Zinn J. (2003). Mindfulness-based interventions in context: past, present, and future. Clin. Psychol. Sci. Pract. 10, 144–156. doi: 10.1093/clipsy.bpg016

[ref47] Kangaslampi S. Peltonen K. (2022). Mechanisms of change in psychological interventions for posttraumatic stress symptoms: a systematic review with recommendations. Curr. Psychol. 41, 258–275. doi: 10.1007/s12144-019-00478-5

[ref48] Kiken L. G. Garland E. L. Bluth K. Palsson O. S. Gaylord S. A. (2015). From a state to a trait: trajectories of state mindfulness in meditation during intervention predict changes in trait mindfulness. Personal. Individ. Differ. 81, 41–46. doi: 10.1016/j.paid.2014.12.044, 25914434 PMC4404745

[ref49] Kimbrough E. Magyari T. Langenberg P. Chesney M. Berman B. (2010). Mindfulness intervention for child abuse survivors. J. Clin. Psychol. 66, 17–33. doi: 10.1002/jclp.20624 19998425

[ref50] Kirk U. Axelsen J. L. (2020). Heart rate variability is enhanced during mindfulness practice: a randomized controlled trial involving a 10-day online-based mindfulness intervention. PLoS One 15:e0243488. doi: 10.1371/journal.pone.0243488, 33332403 PMC7746169

[ref51] Kirlic N. Cohen Z. P. Singh M. K. (2020). Is there an ace up our sleeve? A review of interventions and strategies for addressing behavioral and neurobiological effects of adverse childhood experiences in youth. Advers. Resil. Sci. 1, 5–28. doi: 10.1007/s42844-020-00001-x, 34278327 PMC8281391

[ref52] Kok B. E. Fredrickson B. L. (2010). Upward spirals of the heart: autonomic flexibility, as indexed by vagal tone, reciprocally and prospectively predicts positive emotions and social connectedness. Biol. Psychol. 85, 432–436. doi: 10.1016/j.biopsycho.2010.09.005, 20851735 PMC3122270

[ref53] Lazarus R. S. (1991). Cognition and motivation in emotion. Am. Psychol. 46, 819–834. doi: 10.1037/0003-066X.46.8.819 2048794

[ref54] Lehrer P. M. Gevirtz R. (2014). Heart rate variability biofeedback: how and why does it work? Front. Psychol. 5:756. doi: 10.3389/fpsyg.2014.00756, 25101026 PMC4104929

[ref55] Leyland A. Rowse G. Emerson L.-M. (2019). Experimental effects of mindfulness inductions on self-regulation: systematic review and meta-analysis. Emotion 19, 108–122. doi: 10.1037/emo0000425, 29578742

[ref9003] Li C. Chang Q. Zhang J. Chai W. (2018). Effects of slow breathing rate on heart rate variability and arterial baroreflex sensitivity in essential hypertension. Medicine, 97, e0639. doi: 10.1097/MD.0000000000010639 29718876 PMC6392805

[ref56] Lindahl J. R. Fisher N. E. Cooper D. J. Rosen R. K. Britton W. B. (2017). The varieties of contemplative experience: a mixed-methods study of meditation-related challenges in Western Buddhists. PLoS One 12:e0176239. doi: 10.1371/journal.pone.0176239, 28542181 PMC5443484

[ref57] Lupien S. J. McEwen B. S. Gunnar M. R. Heim C. (2009). Effects of stress throughout the lifespan on the brain, behaviour and cognition. Nat. Rev. Neurosci. 10, 434–445. doi: 10.1038/nrn2639, 19401723

[ref58] Malinowski P. (2013). Neural mechanisms of attentional control in mindfulness meditation. Front. Neurosci. 7:8. doi: 10.3389/fnins.2013.00008, 23382709 PMC3563089

[ref59] May A. (2011). Experience-dependent structural plasticity in the adult human brain. Trends Cogn. Sci. 15, 475–482. doi: 10.1016/j.tics.2011.08.002, 21906988

[ref60] McEwen B. S. (2007). Physiology and neurobiology of stress and adaptation: central role of the brain. Physiol. Rev. 87, 873–904. doi: 10.1152/physrev.00041.2006, 17615391

[ref61] McEwen B. S. (2012). Brain on stress: how the social environment gets under the skin. Proc. Natl. Acad. Sci. 109, 17180–17185. doi: 10.1073/pnas.1121254109, 23045648 PMC3477378

[ref62] McLaughlin K. A. Sheridan M. A. Alves S. Mendes W. B. (2014). Child maltreatment and autonomic nervous system reactivity: identifying dysregulated stress reactivity patterns by using the biopsychosocial model of challenge and threat. Psychosom. Med. 76, 538–546. doi: 10.1097/PSY.0000000000000098, 25170753 PMC4163065

[ref63] McLaughlin K. A. Sheridan M. A. Gold A. L. Duys A. Lambert H. K. Peverill M. . (2016). Maltreatment exposure, brain structure, and fear conditioning in children and adolescents. Neuropsychopharmacology 41, 1956–1964. doi: 10.1038/npp.2015.365, 26677946 PMC4908632

[ref64] Morton M. L. Helminen E. C. Felver J. C. (2020). A systematic review of mindfulness interventions on psychophysiological responses to acute stress. Mindfulness 11, 2039–2054. doi: 10.1007/s12671-020-01386-7

[ref65] Moyes E. Nutman G. Mirman J. H. (2022). The efficacy of targeted mindfulness-based interventions for improving mental health and cognition among youth and adults with ACE histories: a systematic mixed studies review. J. Child Adolesc. Trauma 15, 1165–1177. doi: 10.1007/s40653-022-00454-5, 36439656 PMC9684378

[ref66] Natarajan A. (2023). Heart rate variability during mindful breathing meditation. Front. Physiol. 13:1017350. doi: 10.3389/fphys.2022.1017350, 36756034 PMC9899909

[ref9002] Nusslock R. Miller G. E. (2016). Early-Life Adversity and Physical and Emotional Health Across the Lifespan: A Neuroimmune Network Hypothesis. Biological Psychiatry, 80, 23–32. doi: 10.1016/j.biopsych.2015.05.017 26166230 PMC4670279

[ref67] O’Leary K. O’Neill S. Dockray S. (2016). A systematic review of the effects of mindfulness interventions on cortisol. J. Health Psychol. 21, 2108–2121. doi: 10.1177/1359105315569095, 25673371

[ref68] Ortiz R. Sibinga E. M. (2017). The role of mindfulness in reducing the adverse effects of childhood stress and trauma. Children 4:16. doi: 10.3390/children4030016, 28264496 PMC5368427

[ref69] Peressutti C. Martín-González J. M. García-Manso J. M. (2011). Heart rate variability behavior at different stages of practice in Zen meditation: a study of the system dynamics using multiresolution analysis. Rev. Andal. Med. Deporte 4, 58–62. doi: 10.1016/j.ijcard.2009.06.058

[ref70] Pitman R. K. Rasmusson A. M. Koenen K. C. Shin L. M. Orr S. P. Gilbertson M. W. . (2012). Biological studies of post-traumatic stress disorder. Nat. Rev. Neurosci. 13, 769–787. doi: 10.1038/nrn3339, 23047775 PMC4951157

[ref71] Pole N. (2007). The psychophysiology of posttraumatic stress disorder: a meta-analysis. Psychol. Bull. 133, 725–746. doi: 10.1037/0033-2909.133.5.725, 17723027

[ref72] Porges S. W. Doussard-Roosevelt J. A. Maiti A. K. (1994). Vagal tone and the physiological regulation of emotion. Monogr. Soc. Res. Child Dev. 59, 167–186. doi: 10.2307/1166144, 7984159

[ref73] Rodriguez-Larios J. Faber P. Achermann P. Tei S. Alaerts K. (2020). From thoughtless awareness to effortful cognition: alpha - theta cross-frequency dynamics in experienced meditators during meditation, rest and arithmetic. bioRxiv:5935. doi: 10.1101/2020.01.14.905935 PMC7096392 32214173

[ref74] Santorelli S.F. Kabat-Zinn J. Blacker M. Meleo-Meyer F. Koerbel L. (2017). Mindfulness-based stress reduction (MBSR) authorized curriculum guide. Center for mindfulness in medicine, health care, and society (CFM). University of Massachusetts Medical School.

[ref75] Schmitz M. Back S. N. Seitz K. I. Harbrecht N. K. Streckert L. Schulz A. . (2023). The impact of traumatic childhood experiences on interoception: disregarding one’s own body. Borderline Personal. Disord. Emot. Dysregul. 10:5. doi: 10.1186/s40479-023-00212-5, 36788573 PMC9930318

[ref76] Schnyder U. Ehlers A. Elbert T. Foa E. B. Gersons B. P. R. Resick P. A. . (2015). Psychotherapies for PTSD: what do they have in common? Eur. J. Psychotraumatol. 6:8186. doi: 10.3402/ejpt.v6.28186 PMC4541077 26290178

[ref77] Schumer M. C. Lindsay E. K. Creswell J. D. (2018). Brief mindfulness training for negative affectivity: a systematic review and meta-analysis. J. Consult. Clin. Psychol. 86, 569–583. doi: 10.1037/ccp0000324, 29939051 PMC6441958

[ref78] Shapero B. G. Greenberg J. Pedrelli P. de Jong M. Desbordes G. (2018). Mindfulness-based interventions in psychiatry. Focus 16, 32–39. doi: 10.1176/appi.focus.20170039, 29599651 PMC5870875

[ref79] Shapiro S. L. Carlson L. E. Astin J. A. Freedman B. (2006). Mechanisms of mindfulness. J. Clin. Psychol. 62, 373–386. doi: 10.1002/jclp.20237, 16385481

[ref80] Sheffler J. L. Piazza J. R. Quinn J. M. Sachs-Ericsson N. J. Stanley I. H. (2019). Adverse childhood experiences and coping strategies: identifying pathways to resiliency in adulthood. Anxiety Stress Coping 32, 594–609. doi: 10.1080/10615806.2019.1638699, 31288568 PMC6824267

[ref81] Sheffler J. L. Stanley I. Sachs-Ericsson N. (2020). “ACEs and mental health outcomes,” in Adverse Childhood Experiences, (San Diego, CA: Academic Press), 47–69.

[ref82] Spidel A. Daigneault I. Kealy D. Lecomte T. (2019). Acceptance and commitment therapy for psychosis and trauma: investigating links between trauma severity, attachment and outcome. Behav. Cogn. Psychother. 47, 230–243. doi: 10.1017/S1352465818000413, 30012233

[ref83] Spidel A. Lecomte T. Kealy D. Daigneault I. (2018). Acceptance and commitment therapy for psychosis and trauma: improvement in psychiatric symptoms, emotion regulation, and treatment compliance following a brief group intervention. Psychol. Psychother. 91, 248–261. doi: 10.1111/papt.12159, 28976056

[ref84] Swedo E. A. (2023). Prevalence of adverse childhood experiences among U.S. adults — behavioral risk factor surveillance system, 2011–2020. MMWR Morb. Mortal Wkly. Rep. 72, 707–715. doi: 10.15585/mmwr.mm7226a2, 37384554 PMC10328489

[ref85] Sweeney A. Filson B. Kennedy A. Collinson L. Gillard S. (2018). A paradigm shift: relationships in trauma-informed mental health services. BJPsych Adv. 24, 319–333. doi: 10.1192/bja.2018.29, 30174829 PMC6088388

[ref86] Tang Y.-Y. Holzel B. Posner M. (2015). The neuroscience of mindfulness meditation. Nat. Rev. Neurosci. 16, 213–225. doi: 10.1038/nrn3916 25783612

[ref87] Taren A. A. Gianaros P. J. Greco C. M. Lindsay E. K. Fairgrieve A. Brown K. W. . (2015). Mindfulness meditation training alters stress-related amygdala resting state functional connectivity: a randomized controlled trial. Soc. Cogn. Affect. Neurosci. 10, 1758–1768. doi: 10.1093/scan/nsv066, 26048176 PMC4666115

[ref88] Thayer J. F. Hansen A. L. Saus-Rose E. Johnsen B. H. (2009). Heart rate variability, prefrontal neural function, and cognitive performance: the neurovisceral integration perspective on self-regulation, adaptation, and health. Annals Behav. Med. 37, 141–153. doi: 10.1007/s12160-009-9101-z, 19424767

[ref89] Thayer J. F. Lane R. D. (2000). A model of neurovisceral integration in emotion regulation and dysregulation. J. Affect. Disord. 61, 201–216. doi: 10.1016/S0165-0327(00)00338-4, 11163422

[ref90] Van Dam N. T. van Vugt M. K. Vago D. R. Schmalzl L. Saron C. D. Olendzki A. . (2018). Mind the hype: a critical evaluation and prescriptive agenda for research on mindfulness and meditation. Perspect. Psychol. Sci. 13, 36–61. doi: 10.1177/1745691617709589, 29016274 PMC5758421

[ref91] Wang S.-Z. Li S. Xu X.-Y. Lin G.-P. Shao L. Zhao Y. . (2010). Effect of slow abdominal breathing combined with biofeedback on blood pressure and heart rate variability in prehypertension. J. Altern. Complement. Med. 16, 1039–1045. doi: 10.1089/acm.2009.0577 20954960

[ref92] Wang X. Pei J. Hu X. (2020). The brain-heart connection in Takotsubo syndrome: the central nervous system, sympathetic nervous system, and catecholamine overload. Cardiol. Res. Pract. 2020:4150291. doi: 10.1155/2020/4150291 32211202 PMC7085406

[ref93] Waxenbaum J.A. Reddy V. Das J.M. (2025) “Anatomy, autonomic nervous system,” StatPearls. StatPearls Publishing. (Treasure Island, FL: StatPearls Publishing). Available online at: https://www.ncbi.nlm.nih.gov/sites/books/NBK539845/ (Accessed December 20, 2025). 30969667

[ref94] Wei G.-X. Li Y. F. Yue X. L. Ma X. Chang Y. K. Yi L. Y. . (2016). Tai chi Chuan modulates heart rate variability during abdominal breathing in elderly adults. PsyCh Journal 5, 69–77. doi: 10.1002/pchj.105, 26377754

[ref95] Williams M. Honan C. Skromanis S. Sanderson B. Matthews A. J. (2024). Psychological and attentional outcomes following acute mindfulness induction among high anxiety individuals: a systematic review and meta-analysis. J. Psychiatr. Res. 170, 361–374. doi: 10.1016/j.jpsychires.2023.12.009, 38215647

[ref96] Winzeler K. Voellmin A. Hug E. Kirmse U. Helmig S. Princip M. . (2017). Adverse childhood experiences and autonomic regulation in response to acute stress: the role of the sympathetic and parasympathetic nervous systems. Anxiety Stress Coping 30, 145–154. doi: 10.1080/10615806.2016.1238076, 27653030

[ref97] Zainal N. H. Tan H. H. Hong R. Y. S. Newman M. G. (2024). Testing the efficacy of a brief, self-guided mindfulness ecological momentary intervention on emotion regulation and self-compassion in social anxiety disorder: randomized controlled trial. JMIR Mental Health 11:e53712. doi: 10.2196/53712, 38640015 PMC11069101

[ref98] Zhang D. Lee E. K. P. Mak E. C. W. Ho C. Y. Wong S. Y. S. (2021). Mindfulness-based interventions: an overall review. Br. Med. Bull. 138:ldab005. doi: 10.1093/bmb/ldab005, 33884400 PMC8083197

[ref99] Zhu J. Wekerle C. Lanius R. Frewen P. (2019). Trauma- and stressor-related history and symptoms predict distress experienced during a brief mindfulness meditation sitting: moving toward trauma-informed care in mindfulness-based therapy. Mindfulness 10, 1985–1996. doi: 10.1007/s12671-019-01173-z

